# S07-3 The health promoting sports club model: intervention theory design

**DOI:** 10.1093/eurpub/ckac093.036

**Published:** 2022-08-29

**Authors:** Stacey Johnson

**Affiliations:** Université Côte d'Azur, LAMHESS, France

**Keywords:** health promotion, sports club, settings-based approach, intervention framework, strategies

## Abstract

**Background:**

Sports clubs have been acknowledged as health promoting settings by researchers and policymakers. Limited research has linked the health promoting sports club (HPSC) concept with evidence-driven strategies to provide sports clubs a framework to develop health promotion interventions. As implementation science insists on the creation of theoretically grounded interventions, the objective of this work was to provide sports clubs an evidence-driven intervention framework to implement health promotion in sports clubs.

**Methods:**

An iterative qualitative process in three steps was undertaken to accomplish the objective: (1) adaptation from the HPSC concept to create the HPSC model, (2) reformulation of published evidence-driven guidelines into implementable intervention components (ICs) and (3) merging of the model with the ICs to provide an evidence-based intervention framework for sports clubs. The research team first defined the various elements of the model and formulated ICs, then three groups (French sport students, French experts and Swedish experts) classified the ICs into the HPSC model. In order to retain classification, at least a 2 group agreement was required.

**Results:**

To aid with theory selection, the research team drafted 5 indicators to consider sports clubs as a health promoting setting. Guided by these indicators, the theoretical ‘HPSC concept' was chosen as the basis to define and create the ‘HPSC model', which defines three sports club levels (club, directors, coaches) and four health determinants (organizational, social, environmental, economic) per level. Published guidelines from two literature reviews were used to develop 14 strategies with 55 intervention components. IC categorization by the three focus groups included: 79 classifications at the club level, 67 classifications at the director level and the coaching level retained 48 classifications.

**Conclusions:**

The theoretical HPSC model and designed framework are a starting point to plan, select and deliver interventions to increase HP efforts by stakeholders in several ways: (1) clubs can apply strategies based on goals, (2) clubs can target specific levels with corresponding ICs or (3) ICs can be employed to affect particular determinants of health.

